# Coordination-cage binding and catalysed hydrolysis of organophosphorus chemical warfare agent simulants†

**DOI:** 10.1039/d4ra04705b

**Published:** 2024-08-19

**Authors:** Burin Sudittapong, Christopher G. P. Taylor, James Williams, Rebecca J. Griffiths, Jennifer R. Hiscock, Michael D. Ward

**Affiliations:** a Department of Chemistry, University of Warwick Coventry CV4 7AL UK m.d.ward@warwick.ac.uk; b School of Chemistry and Forensic Science, University of Kent Canterbury CT2 7NH UK

## Abstract

The use of organophosphorus chemical warfare agents still remains an ongoing global threat. Here we investigate the binding of small-molecule organic guests including phosphate esters, sulfonate esters, carbonate esters and a sulfite ester – some of which act as simulants for organophosphorus chemical warfare agents – in the cavity of a water-soluble coordination cage. For several of these guest species, binding constants in the range 10^2^ to 10^3^ M^−1^ were determined in water/DMSO (98 : 2 v/v) solution, through a combination of fluorescence and ^1^H NMR spectroscopy, and subsequent fitting of titration data to a 1 : 1 binding isotherm model. For three cage/guest complexes crystallographic structure determinations were possible: in two cases (with guests phenyl methanesulfonate and phenyl propyl carbonate) the guest lies inside the cavity, forming a range of CH⋯O hydrogen-bonding interactions with the cage interior surface involving CH groups on the cationic cage surface that act as H-bond donors and O atoms on the guests that act as H-bond acceptors. In a third case, with the guest 4-nitrophenyl-methanesulfonate, the guest lies in the spaces outside a cage cavity between cages and forms weak CH⋯O interactions with the cage exterior surface: the cavity is occupied by a network of H-bonded water molecules, though this guest does show cavity binding in solution. For the isomeric guests 4-nitrophenyl-methanesulfonate and 4-nitrophenyl methyl sulfite, hydrolysis in water/DMSO (98 : 2 v/v) could be monitored colorimetrically *via* appearance of the 4-nitrophenolate anion; both showed accelerated hydrolysis rates in the presence of the host cage with second-order rate constants for the catalysed reactions in the range 10^−3^ to 10^−2^ M^−1^ s^−1^ at pH 9. The typical rate dependence on external pH and the increased reaction rates when chloride ions are present (which can bind inside the cavity and displace other cavity-bound guests) imply that the catalysed reaction actually occurs at the external surface of the cage rather than inside the cavity.

## Abstract 2

Simulants of organophosphate chemical warfare agents can bind in the cavity of an octanuclear coordination cage (the figure shows a carbonate-based guest), resulting in some cases in catalysed hydrolytic decomposition.

## Graphical abstract

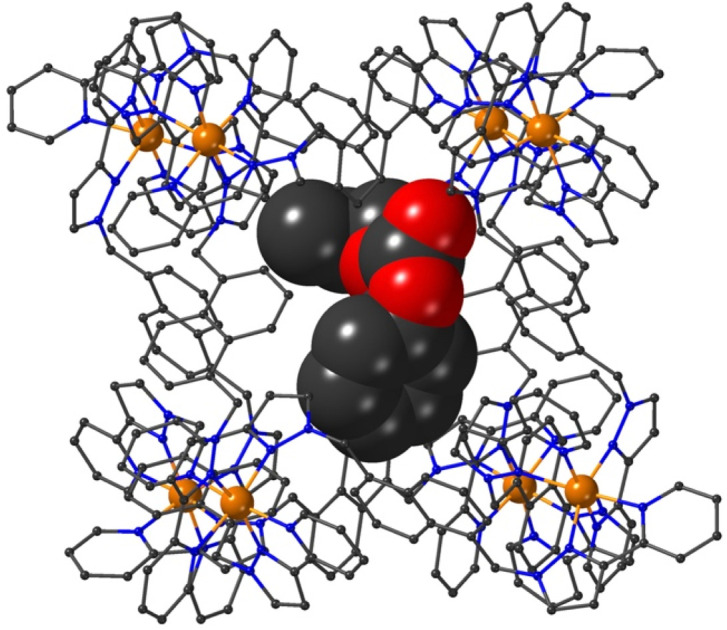

## Introduction

**Fig. 1:**
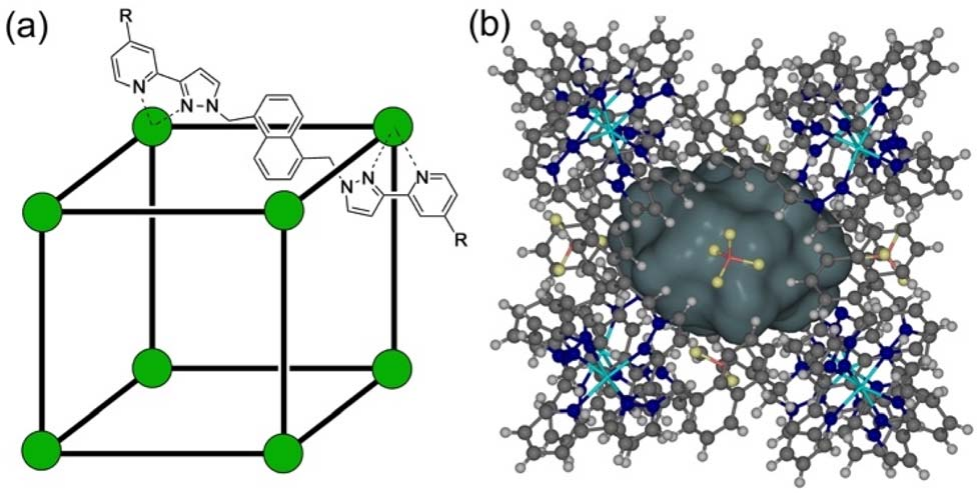
(a) Cartoon illustrating the cubic host cage [M_8_L_12_]^16+^, abbreviated as H (R = H), emphasising the cubic array of Co(ii) ions and the disposition of one bridging ligand; and its derivatives bearing substituents at the twenty-four externally-directed pyridyl C^4^ positions H^W^ (R = CH_2_OH), and H^PEG^ [R = –(CH_2_OCH_2_)_3_CH_2_OMe]. (b) A view of the complete cage structure, highlighting the guest binding cavity space (*V* = 409 Å^3^).

As part of ongoing studies into (i) the host–guest chemistry and catalysis properties of a family of coordination cages,^1^ we report here the interactions of our octanuclear, approximately cubic, coordination cage H^w^ (Fig. 1) with a variety of small molecules that have been used as simulants for chemicīal warfare agents (CWAs).^2^ The ability of self-assembled coordination cages to bind small-molecule guests and thereby act as sensors for the presence of these guests, or as catalysts for enhanced reactivity of the bound guests, are well studied:^3,4^ and CWA simulants (among other toxic species) are obviously appealing targets for such studies.

CWAs are a pernicious category of weaponised substances engineered to inflict harm or death. They are defined as “chemicals intended for use in military operations to kill, seriously injure, or incapacitate people due to their physiological effects”.^5^ There are seven different CWA sub-categories: (i) nerve (organophosphorus – OP) agents, which target the enzyme acetylcholinesterase; (ii) blister agents, which cause severe skin, eye and mucosal pain and irritation; (iii) blood agents, that affect the body by being absorbed into the blood; (iv) incapacitating agents: (v) choking agents; (vi) vomiting agents and; (vii) tear agents. The combined threat of CWAs and their potential deployment in conflicts and terrorist activities highlights the urgent necessity for rapid and reliable detection methods, as well as effective neutralisation techniques.^6^ These protective measures have emerged as a critical area of research in recent times; for obvious reasons such research uses CWA simulants, which replicate key structural and functional group properties of CWAs whilst being much less toxic, enabling safe experimentation within legal restrictions.

The application of supramolecular chemistry to the detection and neutralisation of CWAs and related toxic substrates has been extensively explored. The key process to make these applications work is binding of the substrate to a host molecule. For detection purposes binding needs to be strong enough to generate detectable amounts of the host/guest complex at the low concentrations of host and guest that are likely to be used: fluorescence-based responses are normally measured in the μM concentration domain, for example, requiring commensurately strong guest binding. For neutralisation/catalysed destruction of a substrate a high binding constant may not be necessary: even if only a small fraction of substrate is bound at any time, a fast catalysed reaction can still neutralise substrate on a useful timescale. Whatever the strength, guest binding is driven primarily by the usual weak interactions that are the toolbox of supramolecular chemistry – the hydrophobic effect, induced dipoles, π–π interactions, charge transfer, and hydrogen bonding. Leveraging these interactions, supramolecular chemists have investigated a range of host structures for their capacity to accommodate and catalyse the degradation of CWAs.^5,6^ These hosts include cucurbiturils, cyclodextrins, calixarenes, metal–organic frameworks, and coordination cages, all of which have shown efficacy in accommodating and catalysing degradation of CWAs and their simulants.^6^

We have previously demonstrated that the octanuclear coordination cage H (Fig. 1) binds alkyl phosphonates (simple simulants of nerve/OP CWAs) within its central cavity in water. This process, which is driven by the hydrophobic effect, generates an optical response in the form of partial quenching of the cage fluorescence (arising from the naphthyl groups in the ligands).^7^ In addition, a next-generation cage H^w^, demonstrating increased water solubility *via* the presence of additional external hydroxymethyl substituents,^8^ was found to form a 1 : 1 complex with the guest ‘dichlorvos’ (2,2-dichlorovinyl dimethyl phosphate) – an organophosphate insecticide – bound inside the cage cavity. However, although the presence of the cage was found to increase the hydrolysis rate of dichlorvos in weakly basic aqueous solution, the catalysis was found to occur at the external surface of the cage rather than inside the cavity.^9^

Interestingly the OP CWA sarin (*O*-isopropyl methylphosphonofluoridate) demonstrated an increase in its solution half-life upon binding to a PEG-ylated (and hence water-solubilised) host cage H^PEG^ (Fig. 1), implying that cavity binding results in protection of the cavity-bound guest from the hydroxide ions surrounding the cage in aqueous solution.^10^ This is an exact inversion of the conventional catalysis mechanism for reactions involving cage binding, which involve the cavity-bound guest undergoing accelerated hydrolysis due to the high local concentration of hydroxide ions that accumulate around the 16+ cage surface due to ion-pairing effects.^1,11^

**Fig. 2:**
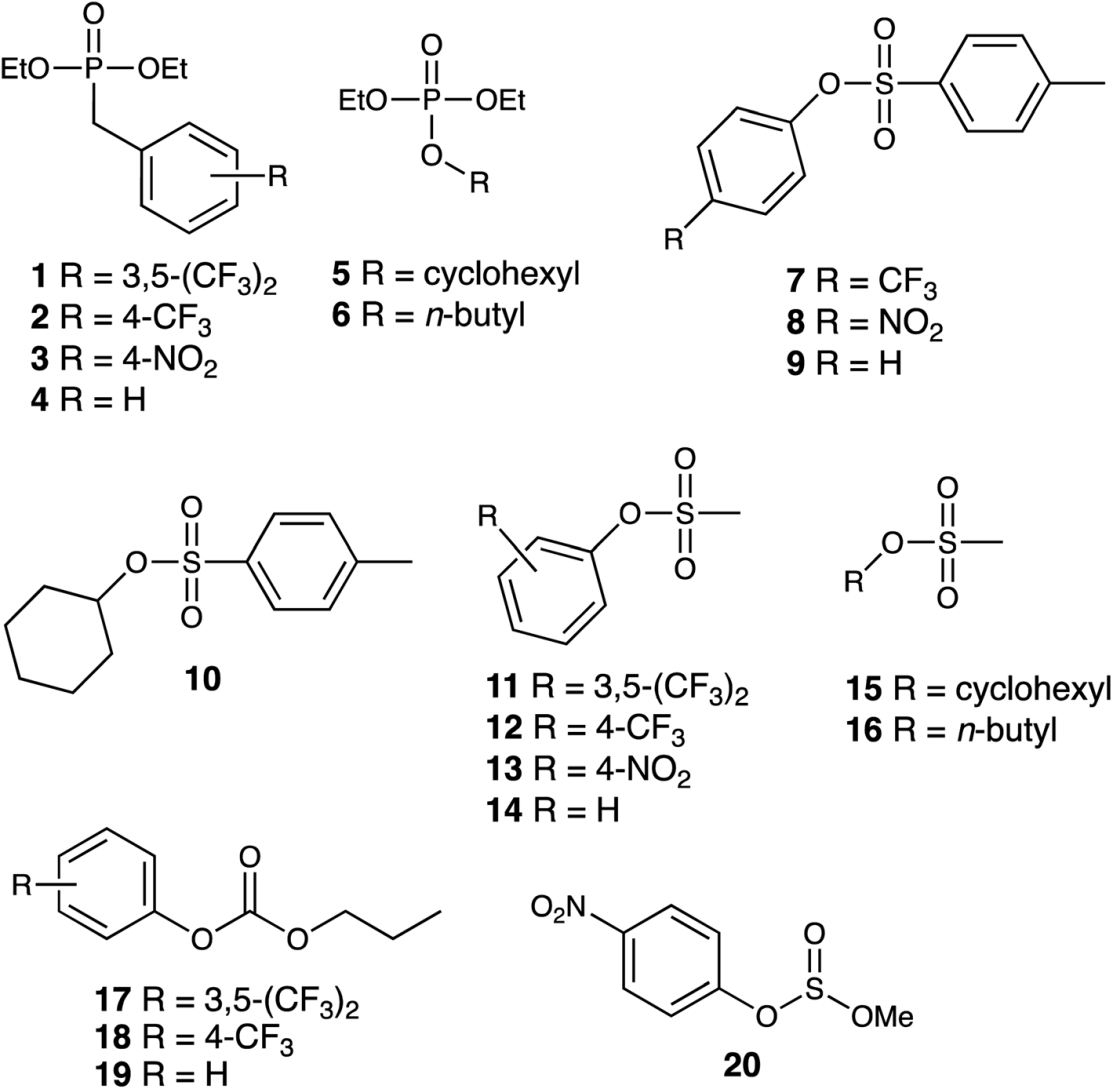
Structural formulae of the series of potential guests investigated.

We report here investigations into the interaction of the set of guests shown in Fig. 2 (which includes examples of some simple OP CWA simulants and consists of a series of phosphate, sulfonate, carbonate and sulfite esters) with the host cage H^w^ in a predominantly aqueous solvent (water : DMSO, 98 : 2, v/v): the small admixture of DMSO is necessary due to the solubility limitations of these guests in pure water. The use of a (predominantly) aqueous solvent system is relevant to any conceivable real-world detection or remediation of genuine CWAs that might occur. It poses challenges in terms of significant solubility limitations of both host and guests, but has the substantial benefit that the hydrophobic effect can be expected to contribute substantially to binding of guests such as these: we know from previous work that water provides far higher binding constants for small organic guests inside host cages of this type than polar organic solvents do for exactly this reason.^1,8^ Overall, with a range of guests, we have measured host/guest binding constants; determined some crystal structures of host/guest complexes; and have investigated the acceleration of hydrolysis of two of these guests arising from cage-based catalysis.

## Results and discussion

### Binding constants of guests in the cage cavity

The small-molecule guests investigated are shown in Fig. 2: their relevant metric properties (molecular volumes in Å^3^ and surface areas in Å^2^) are provided in Table 1. The cavity volume of the cubic host cage H^w^ is 409 Å^3^, with an ideal volume for a guest – based on the Rebek 55% rule – being around 225 Å^3^,^12^ although this is an approximation with considerable latitude:^13^ all of these guests (1–20) are in the appropriate size range for one guest to bind comfortably (37–74% of the cavity volume).

**Table 1:** Guest metric parameters (surface area, SA; and volume, *V*), and binding constants *K* (where measurable) in H^w^·BF_4_ in water/DMSO (98 : 2, v/v)

Series	Guest	SA/Å^2^	*V*/Å^3^	*K*/M^−1^
Phosphates	1	331	302	in^a^
	2	294	265	nb^b^
	3	285	256	qm^c^
	4	258	227	nb^b^
	5	287	245	7.4(4) × 10^2^
	6	272	223	4.9(6) × 10^2^
Tosylates	7	285	272	in^a^
	8	282	264	qm^c^
	9	262	236	in^a^
	10	288	255	3.4(7) × 10^2^
Mesylates	11	238	231	nb^b^
	12	210	195	3.8(9) × 10^2^
	13	201	187	qm^c^
	14	183	159	2.2(5) × 10^3^
	15	205	176	qm^c^
	16	184	153	1.3(7) × 10^2^
Carbonates	17	277	260	nb^b^
	18	251	225	in^a^
	19	219	188	1.1(1) × 10^3^, 1.4(1) × 10^3 ^d
Sulfite	20	198	187	1.1(2) × 10^2 ^d

a in = insoluble in the solvent used.

b nb = no binding detected.

c qm = this guest quenches the fluorescence of MAC so the fluorescence displacement assay was unreliable.

d Binding constant measured by ^1^H NMR titration where guest solubility allowed.

**Fig. 3:**
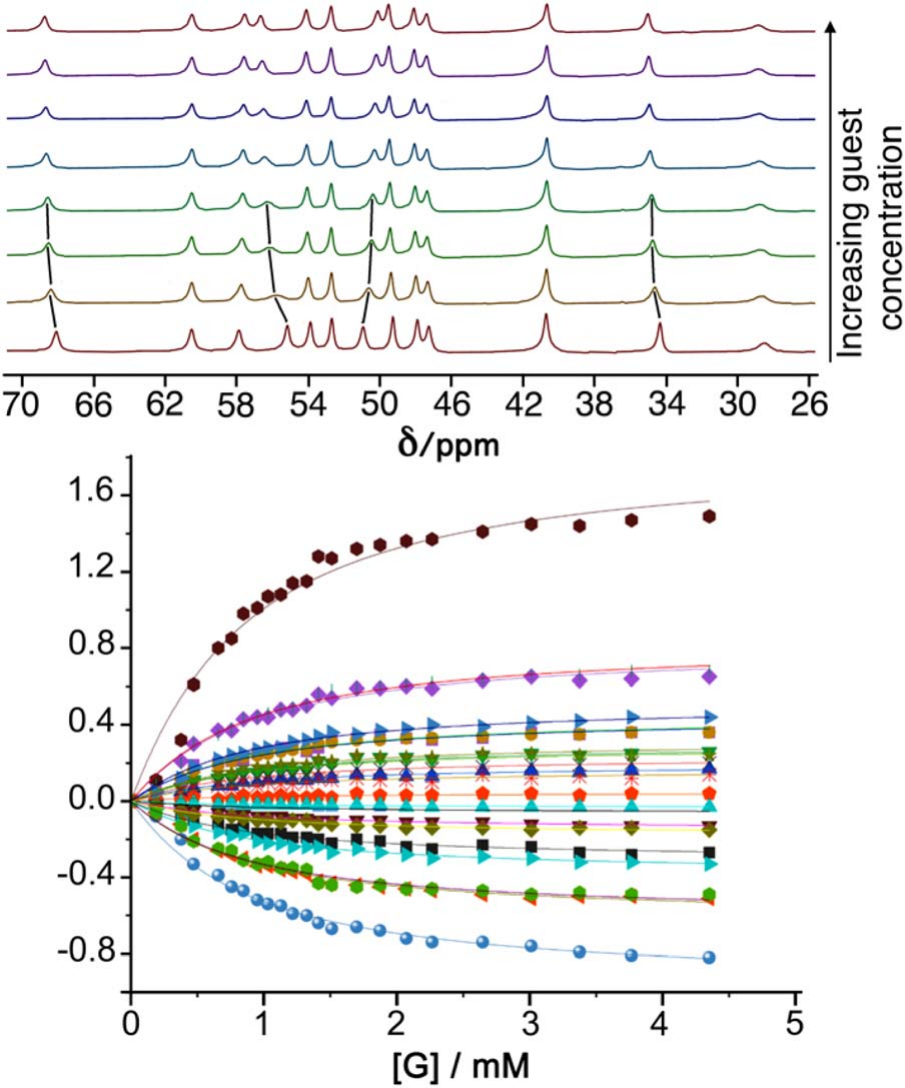
Top: ^1^H NMR spectra of H^W^ in D_2_O/d^6^-DMSO (98 : 2) with increasing concentration of guest 19 (from 0 mM to 4.35 mM) showing the changes in peak position as more guest is added (the movement of some of the signals in the early stages of the titration is highlighted with black lines). Bottom: changes in chemical shifts of signals during the titration, including best fit of the 1 : 1 binding isotherms used to determine the binding constant given in Table 1.

We first measured cage/guest binding constants in water/DMSO (98 : 2) using a previously reported fluorescence-based indicator displacement assay (see ESI† for more details).^8*b*^ The fluorescent guest 4-methyl-7-amino-coumarin (MAC) has its fluorescence quenched by proximity to the Co(ii) ions when binding inside the cage, with a binding constant of *K* = 2.2 × 10^4^ M^−1^ in this mixed solvent determined from a fluorescence quenching titration, which is very similar to the binding constant value observed in pure water.^8*b*^ Stepwise addition of a competing guest in a titration displaces the MAC to an extent dependent on the guest *K* value, and from the rate at which the fluorescence of displaced MAC is restored during the titration, the *K* value for the competing guests can be determined.^8*b*^ Binding constants for those guests that showed evidence of binding and well-behaved binding isotherms are included in Table 1 and are generally in the region 10^2^ to 10^3^ M^−1^, which is fairly typical for many small neutral organic guests in this cage.^1,8^ In some cases these titrations were unsuccessful because of the presence of a nitrophenyl group in the guest which partially quenched the fluorescence of the displaced MAC leading to unreliable results so those values are not included. In two cases, we could obtain 1 : 1 binding constants from NMR titration measurements, but that required guest solubility at the higher concentrations required for NMR spectroscopy measurements so was not widely applicable. Overall, we could obtain 1 : 1 binding constants for 8 of the guests investigated, all lying in a similar range (Table 1). Representative experimental data from an NMR titration experiment with guest 19 are shown in Fig. 3.

### Crystal structures of cage/guest complexes

Crystal structures of some cage/guest complexes were obtained using the ‘crystalline sponge’ methodology.^14^ Here, X-ray quality single crystals of unsubstituted cage H (generated from slow cooling in a solvothermal synthesis) were soaked in concentrated MeOH solutions of the relevant guests overnight, followed by X-ray data collection at the national synchrotron facility. In this way crystal structures of H with the guests phenyl methanesulfonate (14), phenyl propyl carbonate (19), and 4-nitrophenyl methanesulfonate (13) were obtained, of which the first two showed the guest binding inside the cage cavity but the third only displayed guest binding in the space between cage molecules outside the cavity.

**Fig. 4:**
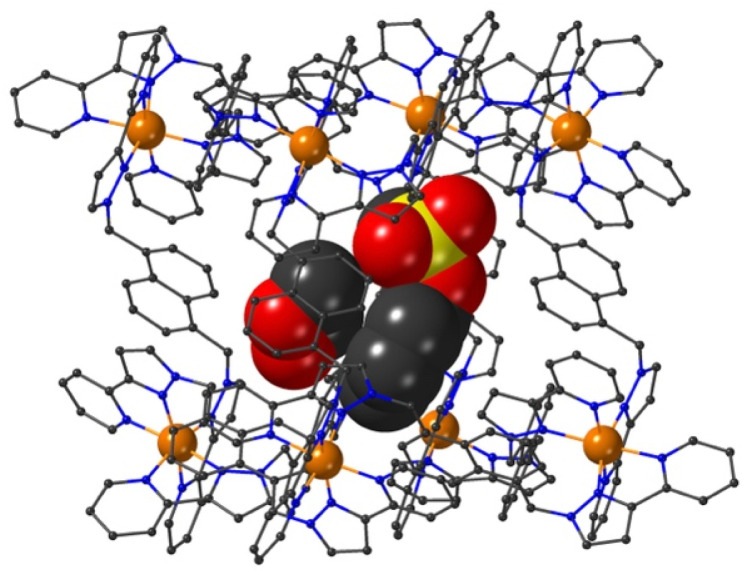
A view based on X-ray crystallography of the host cage H (in wireframe view) containing guests 14 and MeOH (space-filling view): the molecule of 14 is on the right in this view and the two fractional MeOH molecules are on the left.

**Fig. 5:**
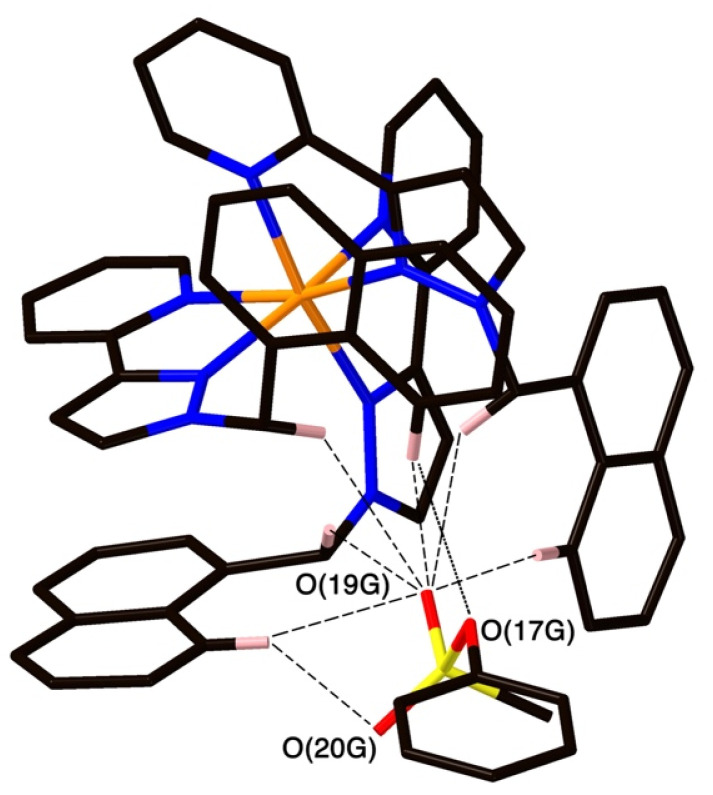
A view of the network of CH⋯O hydrogen-bonding interactions between guest molecule 14 and the convergent array of CH protons (from methylene CH_2_ and naphthyl CH groups) on the interior surface of H around a *fac* tris-chelate metal complex vertex. All O⋯H contacts shown by dashed or dotted lines are <3 Å.

The structure of the cage/guest complex with phenyl methanesulfonate (14) is shown in Fig. 4 and 5. The cavity contains one guest molecule lying on one side and two MeOH molecules on the other; the set of guests exhibits two fold disorder across the crystallographic inversion centre, such that each asymmetric unit contains a phenyl methanesulfonate with a site occupancy of 0.5 and a pair of MeOH molecules with site occupancies of 0.37 and 0.40, giving a total of 1 complete guest 14 and 1.54 MeOH guest molecules in the cavity. Notably we do not see a stacked pair of guests here, which can happen with planar aromatic guests such as coumarins and substituted naphthalenes that can pack together efficiently in the confined space and give high cavity occupancies, approaching 90%.^14^ We attribute the presence of only one guest molecule (plus MeOH molecules) to the non-planar structure of the guest arising from the tetrahedral coordination around the central S atom, which makes efficient stacking in a compact pair more difficult. In addition, we see two additional guest molecules per asymmetric unit (*i.e.*, per half-cage) with site occupancies of 0.47 and 0.73 in spaces between cages and interacting with the exterior surface of the cages;^14^ the aromatic phenyl rings of these exterior guests are also involved in edge-to-face CH⋯π interactions with the cage exterior surface. Overall, therefore, there are 3.4 guests per complete cage, of which 1.0 is cavity bound and the remaining 2.4 are exterior to the cavity.

The two MeOH molecules in the cavity lie sufficiently close to one another that their oxygen atoms O(12S) and O(22S) are separated by 2.75 Å, indicative of an intermolecular OH⋯O hydrogen bond. The cavity-bound phenyl methanesulfonate guest displays numerous H-bonding interactions between the sulfonate unit (where the O atom lone pairs act as H-bond acceptors) and the convergent set of CH proton around the two *fac* tris-chelate vertices of the cage which form H-bond donor pockets.^1,8,13,15^ All three O atoms of the cavity-bound guest are involved in CH⋯O interactions with the surrounding ligand array, but it is atom O(19G) – which projects into the pocket such that it lies only 5.39 Å from the Co(ii) centre Co(4) – which has the largest number of such interactions, with six O⋯H contacts of <3 Å (covering the range 2.45–2.80 Å) involving methylene CH_2_ and naphthyl CH protons: these are highlighted in Fig. 5 with dashed lines. Additional CH⋯O interactions of <3 Å involving interactions of the cage wall with O(17G) and O(20G) are also included in Fig. 5.

**Fig. 6:**
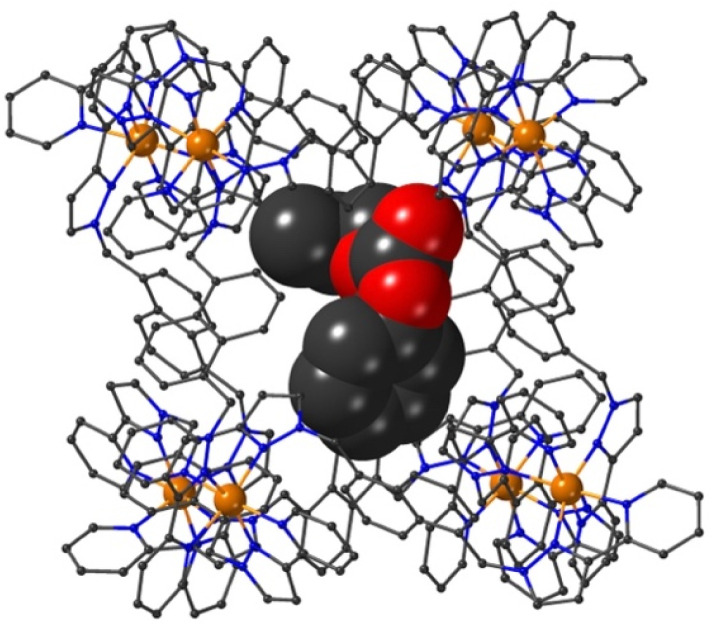
A view based on X-ray crystallography of the host cage H (in wireframe view) containing guests 19 (space-filling view) and MeOH (omitted for clarity).

**Fig. 7:**
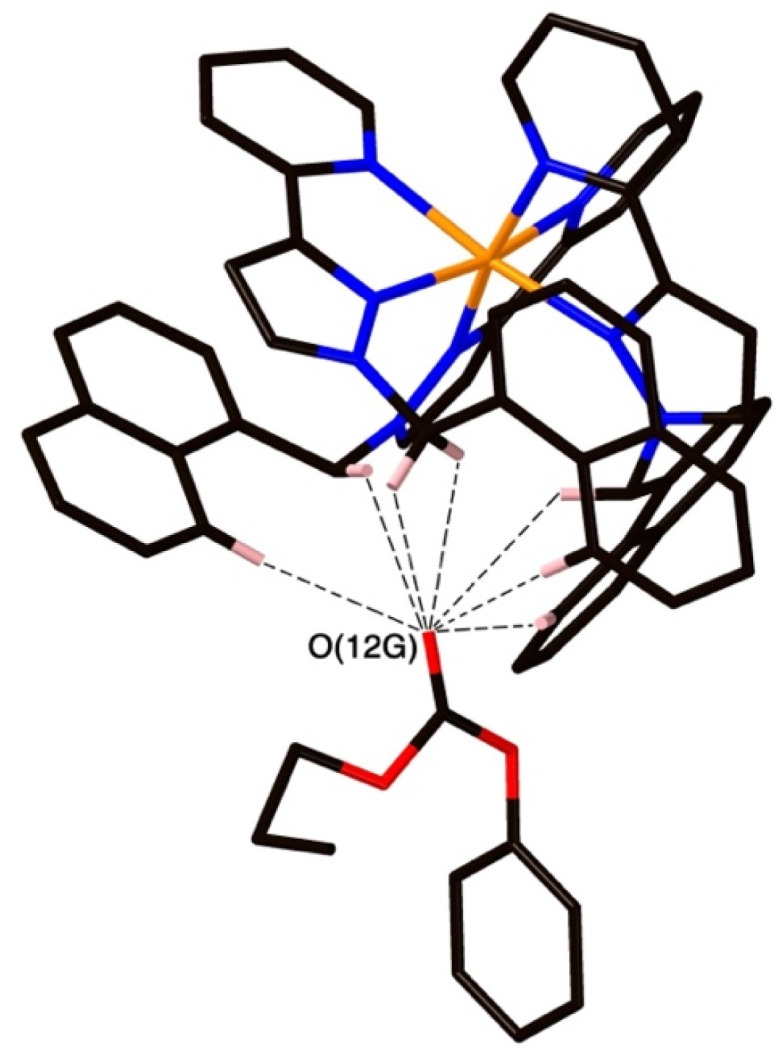
A view of the network of CH⋯O hydrogen-bonding interactions between guest molecule 19 and the convergent array of CH protons (from methylene CH_2_ and naphthyl CH groups) on the interior surface of H around a *fac* tris-chelate metal complex vertex. All O⋯H contacts shown by dashed or dotted lines are <3 Å.

**Fig. 8:**
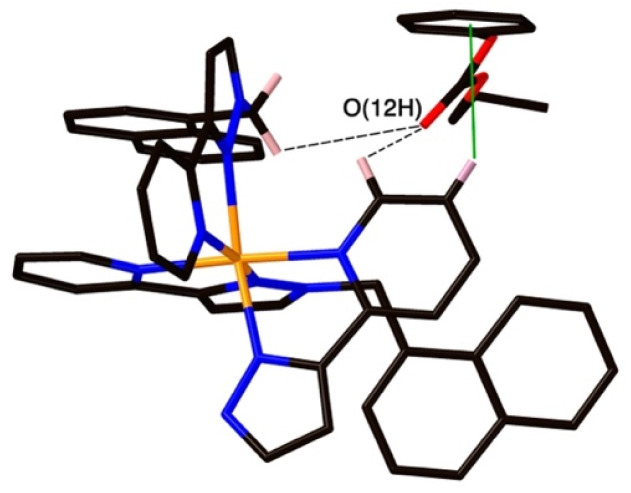
A view of the external guest in the structure of H·19, showing (a) CH⋯O contacts between guest and cage exterior surface whose H⋯O distances are <3 Å (black dashed lines) and (b) a CH–π contact (green line) with a distance of 2.74 Å.

The structure of the cage/guest complex with phenyl propyl carbonate (19) is in Fig. 6–8 (see also ESI† for a view showing thermal ellipsoids). The asymmetric unit of the cage contains a guest 19 in the cavity with site occupancy of 0.35, and one MeOH with site occupancy 0.65; hence, when the twofold disorder across the inversion centre is taken into account, the entire cavity contains 0.7 phenyl propyl carbonate and 1.3 MeOH guests. Binding of the guest 19 in the cavity is facilitated by it adopting a significantly folded conformation, which is different from the more open conformation of the exterior guests which are free of cavity-based steric constraints. One additional phenyl propyl carbonate molecule per asymmetric unit (100% site occupancy) lies outside the cavity in contact with the cage exterior surface *via* weak CH⋯O interactions (Fig. 8), such that overall there are 2.7 guests per complete cage (0.7 internal, 2.0 external). For the cavity-bound guest, it is the C=O oxygen atom O(12G) of each guest which protrudes into one of the H-bond donor pockets around the *fac* tris-chelate vertices [O(12G)⋯Co(4) separation, 5.36 Å; Fig. 6], forming seven CH⋯O interactions of <3 Å involving naphthyl CH and methylene CH_2_ protons, with these non-bonded O⋯H separations in the range 2.47–2.99 Å.

**Fig. 9:**
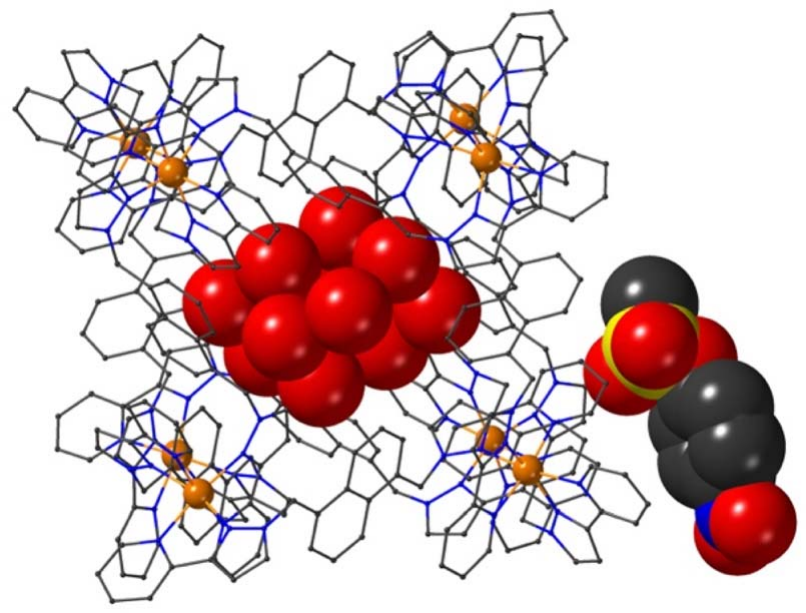
A view based on X-ray crystallography of the host cage H (in wireframe view) containing a network of hydrogen bonded water molecules inside the cavity, and guest 13 outside the cavity. The host cage is shown in wireframe view and the guests inside and outside the cavity are shown as space-filling.

**Fig. 10:**
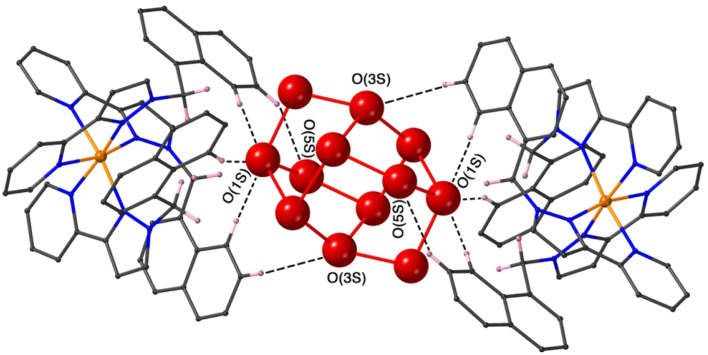
A view based on X-ray crystallography of the water cluster in the cage cavity of the H·13 complex and its interactions with the cage interior surface. O⋯O contacts of <3 Å (indicative of OH⋯O hydrogen-bonding interactions) are shown as red solid lines; CH⋯O contacts of <3 Å involving some of the naphthyl CH protons are shown as black dashed lines. (iii) Catalysed hydrolysis of bound guests.

The final structure is with 4-nitrophenyl-methylsulfonate (13) as guest (Fig. 9 and 10). In this case the guest is taken up into the crystal during the soaking process but lies outside the cavity in the spaces between adjacent cage molecules.^14^ There is one guest 13 per asymmetric unit with a site occupancy of 0.55: a complete cage contains two asymmetric units, hence there are 1.1 guests per cage interacting with the cage exterior surfaces *via* CH⋯O hydrogen bonds, of which shortest is O(31G)⋯H(61B) at 2.35 Å.

The cavity of each cage is occupied by a hydrogen-bonded cluster of 12 water molecules whose total site occupancies (fixed variously at 1.0, 0.75 and 0.5) add up to 7.5H_2_O molecules; the O⋯O separations between adjacent water molecules in the cluster lie in the range 2.60–2.86 Å, indicative of significant HO⋯H hydrogen bonding interactions within the cavity-bound water cluster.^16^ The oxygen atom O(1S), and its symmetry equivalent, lie closest to the H-bond donor pockets situated around the *fac* tris-chelate metal complex vertices (Fig. 10). CH⋯O contacts of <3 Å are shown in Fig. 10 and involve several of the naphthyl CH protons; there are additional CH⋯O contacts of just over 3 Å associated with the methylene CH_2_ protons (not shown in Fig. 10 for clarity). Whilst geometric minutiae associated with this water cluster should not be over-analysed given the evident disorder, basically the water cluster is acting like other organic guests do in respect of interactions with the cage interior surface, and projection into the H-bond donor pocket means that the O(1S)⋯Co separation is 5.74 Å.

Compared to phenyl-methylsulfonate (guest 14) it may be that the additional nitro group on the phenyl ring of 13 causes steric problems with cavity binding, though we should not read too much into this given that the crystals of the cage/guest complexes form under kinetic conditions in the crystal soaking experiments, and do not necessarily represent thermodynamic minima. We have noted before that some guests can be taken up into crystals but bind in spaces between the molecules, even when in solution there is evidence of cavity binding by *e.g.* NMR spectroscopy.^13,14^

### Catalysed hydrolysis of bound guests

For investigation of catalysed hydrolysis of guests from this family we opted for the 4-nitrophenyl substituted guests 8, 13 and 20. These provide, in principle, straightforward reaction monitoring *via* formation of the coloured 4-nitrophenolate anion as a hydrolysis product. The use of UV/vis spectroscopy for the analysis also allows use of relatively low concentrations, which is useful as the poor solubility of many of these guests in water precludes use of the much higher concentrations required for NMR spectroscopy. Of the three guests with 4-nitrophenyl substituents, the sulfonate 8 turned out to be too insoluble to be useful, leaving us focussing our attention on catalysed hydrolysis of the two isomeric guests 20 (4-nitrophenyl methyl sulfite) and 13 (4-nitrophenyl methyl sulfonate). In principle, with 20 as substrate, a hydrolysis reaction could proceed in one of two ways with either methoxide or 4-nitrophenolate as the leaving group: however, the obvious p*K*_a_ difference makes it safe to assume that 4-nitrophenolate formation dominates and is indicative of the overall reaction rate.^17^

**Fig. 11:**
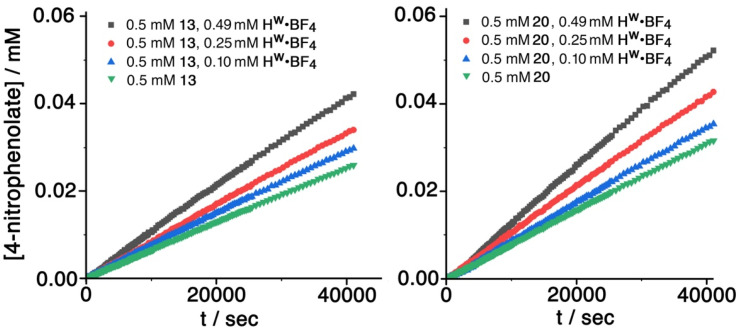
Progress of hydrolysis of 13 and 20 at various concentrations of H^W^·BF_4_ under reaction conditions of 2% DMSO/borate buffer (100 mM, pH 9.0) at 303 K, monitored by the increasing absorbance of the 4-nitrophenolate anion as the reactions progress. Each data set represents the average of three repetitions.

The hydrolysis reactions were conducted in a 2% DMSO/borate buffer (100 mM, pH 9.0) at 303 K, using fixed concentrations of substrate and variable concentrations of cage H^w^ (as its tetrafluoroborate salt, denoted H^w^·BF_4_; and as the anion-exchanged chloride salt, denoted H^w^·Cl) as catalysts. Both guests 13 and 20 exhibited slow background hydrolysis in the absence of any cage catalyst under these conditions with <10% conversion to product after 11 h, and the sulfonate 13 hydrolysing slightly more slowly than the sulfite 20. In the presence of cage H^w^·BF_4_ the reaction rate was significantly increased in both cases, with the increase varying directly with concentration of H^w^·BF_4_, indicating first-order behaviour in catalyst, and giving overall second-order catalysed reaction rate constants of 1.8(2) × 10^−3^ and 2.1(1) × 10^−3^ M^−1^ s^−1^ for the sulfonate 13 and sulfite 20 respectively. These data are summarised in Table 2 and are comparable to what has been observed in other cases of catalysis using this cage: for example we have reported *k*_2_ values in the range 10^−3^ to 10^−2^ M^−1^ s^−1^ for cage-catalysed hydrolysis of diacetyl-fluorescein,^18^ and a range of organophosphate esters under analogous experimental conditions.^9^ Sample experimental measurements with fixed amount of substrate but increasing amounts of cage catalyst for 13 and 20 are shown in Fig. 11; the data in Table 2 is based on this.

**Table 2:** Kinetic data for cage-catalysed hydrolyses of guests 13 and 20 by H^w^·BF_4_ in water/DMSO (98 : 2, v/v)^a^

Guest	[Host]/mM	Initial rate/M s^−1^	*k*_obs_/s^−1^	*k*_2_/M^−1^ s^−1^
0.50 mM 13	0	6.35(1) × 10^−10^	1.27 × 10^−6^	n/a
	0.10	7.32(1) × 10^−10^	1.46 × 10^−6^	1.8(2) × 10^−3^
	0.25	8.43(1) × 10^−10^	1.69 × 10^−6^	
	0.49	1.05(1) × 10^−9^	2.09 × 10^−6^	
0.50 mM 20	0	7.81(1) × 10^−10^	1.56 × 10^−6^	n/a
	0.10	8.83(1) × 10^−10^	1.77 × 10^−6^	2.13(8) × 10^−3^
	0.25	1.05(1) × 10^−9^	2.11 × 10^−6^	
	0.49	1.31(1) × 10^−9^	2.62 × 10^−6^	

a The reaction condition was 2% v/v DMSO in borate buffer (100 mM, pH 9.0) at 303 K. The numbers quoted in parentheses represent errors from the linear fit (initial rate values) or standard deviation based on multiple repeats (*k*_2_ values). Each experiment was performed in triplicate.

Similar experiments conducted under analogous conditions with H^w^·Cl as the catalyst (see ESI†) resulted in a slightly stronger catalytic effect, with second-order catalysed reaction rate constants (Table 3) of 3.9(3) × 10^−3^ and 4.8(2) × 10^−3^ M^−1^ s^−1^ for the sulfonate 13 and sulfite 20, respectively, being in each case approximately double what was observed with H^w^·BF_4_. This implies that the catalysed reaction actually occurs at the cage external surface rather than in the cavity, because the presence of chloride as counter-ion tends to reduce binding constants for guests inside the cage cavity, probably *via* a competitive effect as chloride can also bind inside the cage.^19^ If a reduced fraction of cavity-bound guest leads to a greater reaction rate, then the catalysed reaction must be occurring at the cage external surface, as we have noted in other recent studies of catalysis using diacetylfluorescein,^18^ 4-nitrophenylacetate^18^ and 5-nitro-1,2-benzisoxazole^20^ as substrates.

**Table 3:** Kinetic data for cage-catalysed hydrolyses of guests 13 and 20 by H^w^·Cl in water/DMSO (98 : 2, v/v)^a^

Guest	[Host]/mM	Initial rate/M s^−1^	*k*_obs_/s^−1^	*k*_2_/M^−1^ s^−1^
0.50 mM 13	0.10	8.31(1) × 10^−10^	1.66 × 10^−6^	3.9(3) × 10^−3^
	0.25	1.15(1) × 10^−9^	2.31 × 10^−6^	
	0.49	1.60(1) × 10^−9^	3.20 × 10^−6^	
	1.00	2.38(1) × 10^−9^	4.76 × 10^−6^	
0.50 mM 20	0.10	1.03(1) × 10^−9^	2.06 × 10^−6^	4.8(2) × 10^−3^
	0.25	1.38(1) × 10^−9^	2.76 × 10^−6^	
	0.49	1.96(1) × 10^−9^	3.92 × 10^−6^	
	1.00	3.01(1) × 10^−9^	6.01 × 10^−6^	

a The reaction conditions and calculations for this table are as described for Table 2.

This observation prevents calculation of a *k*_cat_/*k*_uncat_ ratio. If the catalysis of guest hydrolysis occurred inside the cavity, and were therefore linked to the binding constant, then the calculation would be simple. For example for the sulfite guest 20, and using the kinetic data for a catalyst concentration of 0.49 mM, we see an increase in reaction rate of 68% (Table 2). Given the cavity binding constant of 110 M^−1^, and knowing substrate and cage concentrations, we find that around 5% of catalyst is occupied by guest, leading to a catalytic rate enhancement *k*_cat_/*k*_uncat_ of 0.68/0.05 = 14 if catalysis were solely associated with guest binding in the cavity. If however, catalysis is external-surface based then all we can say is that the 95% fraction of guest that is not bound, but can contact the external surface, leads to the rate enhancements reported in Tables 2 and 3: without knowing an association constant between cage and (external) guest we cannot determine *k*_cat_/*k*_uncat._ We note that a standard Michaelis–Menten titration of the type we have used before (to evaluate surface-binding of diacetylfluorescein as substrate^18^) cannot be used: this analysis requires that diacetylfluorescein does not cavity-bind but only interacts with H^w^*via* the external surface, meaning that measurements of catalysis rate *vs.* substrate concentration can be readily interpreted using the Michaelis–Menten model.^18^ Clearly these new substrates 13 and 20 can undergo both cavity-based and external surface binding, of which the latter dominates the catalysis, a more complex situation which is not susceptible to analysis in the same way.

Further evidence of catalysis at the cage exterior surface is provided by the pH dependence of the catalysed reaction rates (Table 4). We found that hydrolysis of the sulfite substrate 20 at pH 10.0 proceeded 7–9 times faster at pH 10 than at pH 9.0 in buffered solutions (see ESI†). This might seem inevitable: but for the cavity-based catalysis of the Kemp elimination (reaction of benzisoxazole with hydroxide ions), the fact that the cage surface is saturated with hydroxide ions due to its 16+ charge – even at modest pH values – meant that increasing the bulk pH of the solution did not increase the local concentration of hydroxide ions surrounding the central cavity. Thus, there was no increase in rate of hydrolysis of the cavity-bound substrate over a wide pH range.^11^ Observation of the expected increase in reaction rate with bulk pH here, however, implies that the reaction is occurring outside the cavity where the substrate can experience the increase in [HO^−^] concentration at the higher pH value, with entirely predictable consequences.

**Table 4:** Kinetic data for cage-catalysed hydrolysis of guest 20 by H^w^·Cl in water/DMSO (98 : 2, v/v) at pH 10^a^

Experiment	Initial rate/M s^−1^	*k*_obs_/s^−1^	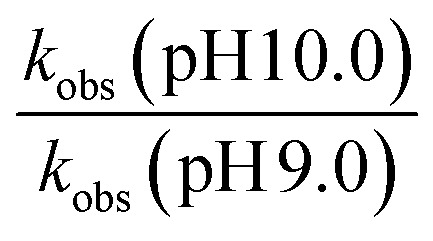
20 (0.35 mM)	5.52(1) × 10^−9^	1.58 × 10^−5^	10.1
20 (0.35 mM), H^W^·Cl (0.1 mM)	6.64(3) × 10^−9^	1.90 × 10^−5^	9.22
20 (0.35 mM), H^W^·Cl (0.5 mM)	1.03(1) × 10^−8^	2.93 × 10^−5^	7.46

a The reaction condition was 2% v/v DMSO in borate buffer (100 mM, pH 10.0) at 303 K. Other conditions are as per Tables 2 and 3 The *k*_obs_ values at pH 9.0 used in the calculation were taken from Table 3.

## Conclusions

This study has reported an examination of binding interactions and catalytic capabilities of the cage H^w^ with some OP CWA simulants. Using a solvent system of DMSO : water (2 : 98%, v/v) we measured a range of binding constants of small CWA simulant guests lying in the range 10^2^ to 10^3^ M^−1^, and three such cage/guest complexes were structurally characterised by X-ray crystallography revealing a range of interactions of guests with both internal and external surfaces of the cage host. The two (isomeric) guests 4-nitrophenyl methanesulfonate (13) and 4-nitrophenyl methyl sulfite (20) both showed an increase in rate of hydrolysis (as determined by colorimetric analysis of the 4-nitrophenolate anion) in the presence of cage H^w^, with the reaction rate increasing linearly with catalyst concentration and second-order rate constants for the catalysed reaction in the range 10^−3^ to 10^−2^ M^−1^ s^−1^. The sensitivity of the catalysis rate constant to the nature of the cage counter ion, and also to the pH of the bulk solution, suggests that – even though the substrates are clearly capable of binding inside the cage cavity – the catalysis actually occurs outside the cavity at the cage exterior surface, where hydrophobic association of cage and substrate brings the substrate molecules into the vicinity of the high local concentration of hydroxide ions that accumulate around the positively-charged cage surface. For practical, real-world applications the main limitation is that pure water is required as the solvent system: however that limitation in this work is a function of the guests used not the cage host, and we have demonstrated elsewhere that catalysed destruction of toxic guests is possible in pure water if the guest is soluble enough.^9^

## Experimental

The cages H^W^·BF_4_ and H^W^·Cl,^8*a*,21^ the unsubstituted cage H used for crystal sponge experiments,^22^ and the collection of CWA simulants used as guests,^2^ were prepared as previously reported.^2^ Other chemicals, reagents, and solvents were purchased from the following commercial sources: Fluorochem; Sigma-Aldrich; Fisher Scientific; and Alfa Aesar, and used as supplied. NMR spectra were recorded on Bruker Avance 300 MHz, Bruker Avance III 400 MHz, Bruker Avance III HD 400 MHz, or Bruker Avance NEO 400 MHz spectrometers at room temperature. Molecular volumes and surface areas were determined from B3LYP 6-31G* DFT calculations implemented in Spartan’06.^23^

### Binding constant determination of MAC

Stock solutions of 4-methyl-7-aminocoumarin (MAC, 0.01 mM) and H^W^ (0.4 mM) were prepared in 98 : 2 water/DMSO (v/v). The titration was performed using 24 samples containing varying ratios of MAC and H^W^. Each sample was prepared by pipetting the appropriate amounts of each solution into each well of a Greiner Bio-One 96-well Microplate, to a total volume of 200 μl per well. The microplate was shaken and incubated at 298 K for 10 minutes before measuring the fluorescence of each well (*λ*_ex_ = 400 nm, *λ*_em_ = 450 nm) using a BMG Labtech ClarioStar microplate reader. The titration was repeated three times, and the datasets were averaged before analysis. A standard 1 : 1 binding isotherm was then fitted to the results using the fitting function in Origin Pro software.^24^

### Guest binding constant measurements by fluorescence displacement assays

Stock solutions of MAC, H^W^ and the guest being evaluated, were combined in varying proportions such that the concentration of MAC was 0.01 mM, the concentration of H^W^ was 0.0574 mM, and the concentration of the guest under evaluated varied from 0 to 4.4 mM in 17 increments [in 98 : 2 water/DMSO (v/v) in all cases]. Each sample was prepared by pipetting the appropriate amounts of the solutions into each well of a Greiner Bio-One 96-well Microplate to a total volume of 100 μl per well. The microplate was shaken and incubated at 298 K for 10 minutes before measuring the fluorescence of each well (*λ*_ex_ = 400 nm, *λ*_em_ = 450 nm) using a BMG Labtech ClarioStar microplate reader. The titration was repeated three times, and the datasets were averaged before analysis. A 1 : 1 binding isotherm was fitted to the results using a calculation taking into account the known binding constant of MAC, as described previously.^8*b*^

### Guest binding constant measurements by NMR titrations

The stock solutions of H^W^ and the guest being evaluated, in D_2_O/d^6^-DMSO (98 : 2 v/v), were combined in different proportions, to a total sample volume of 0.6 ml in a standard NMR tube, to give a total of ≈20 different compositions per experiment with a fixed concentration of H^w^ but varying concentrations of guest. As binding of the two relevant guests was in fast exchange on the NMR timescale, graphs of chemical shift change Δ*δ* for selected host proton signals as a function of guest concentration were fitted to a 1 : 1 binding isotherm using the Origin Pro software.^24^

### Catalysis studies

Stock solutions of host H^W^ (0.8 mM, as either the BF_4_^−^ or the chloride salt) were prepared in 100 mM borate buffer, with pH checked using a Hanna HI 2210 pH meter. Guest solutions (25 mM) were prepared in DMSO. Catalytic studies were performed by adding appropriate amounts of the host, guest, and buffer into each well of a Greiner Bio-One 96-well Microplate, to a total volume of 200 μl per well and a final solvent composition of 2% DMSO/98% water (v/v). The reaction progress at 303 K was monitored by following the absorption of the 4-nitrophenolate anion at 400 nm (*ε* = 1.280 × 10^4^ M^−1^ cm^−1^) using a BMG Labtech ClarioStar microplate reader. Each measurement was repeated three times, and the datasets were averaged before analysis.

### X-ray crystallography

Samples of cage/guest complexes based on the unsubstituted cage H were prepared by prolonged (overnight) immersion of single crystals of H^W^ in concentrated solutions of the guests in MeOH according to the ‘crystalline sponge’ methodology that we have reported previously.^13^ The crystallographic data were acquired using synchrotron radiation at Beamline I19, Diamond Light Source, UK; details of software and methodology used for data reduction, solution and refinement of the structures are as reported previously.^25^ Detailed information on the crystal properties, data collection, and refinement parameters associated with the structure determinations is compiled in Table 5.

**Table 5:** Crystal parameters, data collection and refinement details for the crystal structures

Complex	H·(14)_3.4_	H·(19)_2.7_	H·(13)_1.1_
CCDC number	2366303	2366305	2366304
Formula	C_381.31_H_377.35_B_16_Co_8_F_64_N_72_O_31.73_S_3.39_	C_364.30_H_300.30_B_13.43_Cl_0.67_Co_8_F_53.72_N_72_O_9.40_	C_374.7_H_410.8_B_14_Cl_2_Co_8_F_56_N_73.1_O_44_S_1.1_
Molecular weight	8445.47	7498.1	8435.29
*T*/K	100	100	100
Radiation wavelength/Å	0.6889	0.6889	0.6889
Crystal system	Monoclinic	Monoclinic	Monoclinic
Space group	*C*2/*c*	*C*2/*c*	*C*2/*c*
*a*/Å	33.49900(18)	33.15338(8)	33.1048(4)
*b*/Å	29.42340(18)	29.94428(8)	29.4048(3)
*c*/Å	39.9273(2)	40.69995(12)	40.0356(5)
*β*/°	95.3390(5)	95.6581(2)	96.4162(12)
*V*/Å^−3^	39183.8(4)	40208.18(13)	38728.1(9)
*Z*	4	4	4
*ρ*/g cm^−3^	1.43	1.239	1.447
Crystal size/mm^3^	0.05 × 0.04 × 0.03	0.04 × 0.03 × 0.02	0.09 × 0.08 × 0.07
*μ*/mm^−1^	0.419	0.382	0.425
Data, restraints, parameters	62 330, 6420, 2648	63 895, 10 160, 2562	61 628, 5666, 2375
*R*_int_, *R*_sigma_	0.0730, 0.0679	0.0840, 0.0797	0.0729, 0.0531
Final *R*_1_, w*R*_2_^a^	0.0741, 0.2568	0.0766, 0.2629	0.0926, 0.3330
Largest diff. Peak/hole/e Å^−3^	1.29/−0.92	1.71/−1.05	1.85, −0.94

a The value of *R*_1_ is based on ‘observed’ data with *I* > 2*σ*(*I*); the value of w*R*_2_ is based on all data.

## Data availability

Crystallographic data for the three structures has been deposited at the Cambridge Crystallographic Data Centre (https://www.ccdc.cam.ac.uk) under accession numbers 2366303–2366305.† Additional experimental data not included in the main text can be found in the ESI.†

## Author contributions

B. S. performed the cage syntheses and catalysis studies. C. G. P. T. and J. W. performed the X-ray crystallography. R. J. G. and J. R. H. synthesised the guest molecules. M. D. W. conceived and supervised the project. The manuscript was prepared by M. D. W. and J. R. H.

## Conflicts of interest

There are no conflicts to declare.

## Supplementary Material

RA-014-D4RA04705B-s001

RA-014-D4RA04705B-s002
