# Supplementary material for: Coordination-cage binding and catalysed hydrolysis of organophosphorus chemical warfare agent simulants
Source: RSC Adv. 2024 Aug 19;14(36):26032–42. doi: 10.1039/d4ra04705b (PMC11331485; doi:10.1039/d4ra04705b)
Supplement: RA-014-D4RA04705B-s001 [file RA-014-D4RA04705B-s001.pdf]

This journal is © The Royal Society of Chemistry 2024

Coordination-cage binding and catalysed hydrolysis of organophosphorus chemical warfare agent simulants

Supporting Information

1. Binding isotherm for MAC binding and displacement by carbonate guest 19

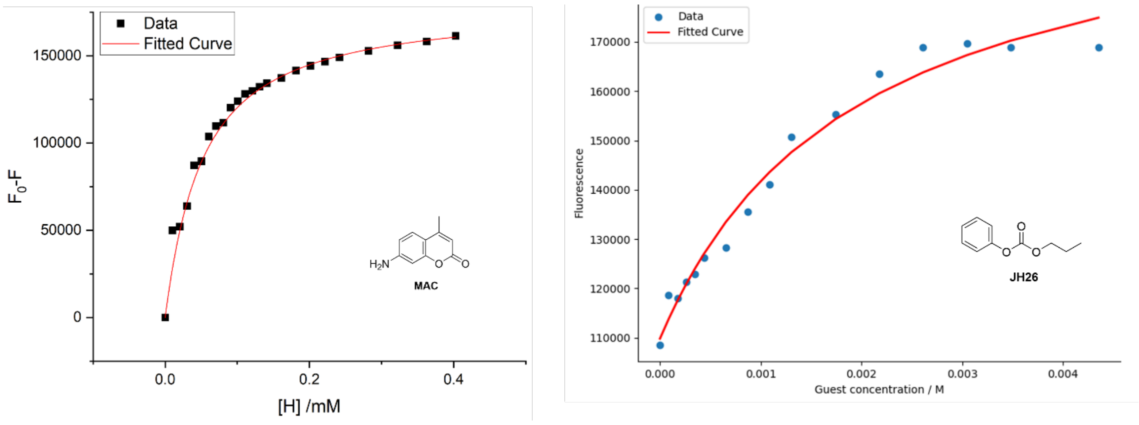

Figure S1. (Left) 1:1 binding isotherm for MAC with $\mathbf{H^W}$ (right) binding isotherm of IDA experiments for **19** with $\mathbf{H^W}$. The fluorescence in both experiments is the emission of MAC measured at 450 nm.

2. Details on the fitting of the 1:1 binding model to the IDA data

In our indicator displacement assay, both the indicator and the guest molecule exhibit a 1:1 binding stoichiometry when they bind with the host molecule.

$$\mathrm{HI} + \mathrm{G} \rightleftharpoons \mathrm{HG} + \mathrm{I} \tag{1}$$

Therefore, in instances of the fluorescent indicator displacement assay where the host and guest molecules are transparent at the observed wavelength, Equations (2) and (3) can be accordingly derived.

$$[\mathrm{H}]_\mathrm{t} = [\mathrm{H}] + \frac{K_\mathrm{G}[\mathrm{H}]}{1 + K_\mathrm{G}[\mathrm{H}]}[\mathrm{G}]_\mathrm{t} + \frac{K_I[\mathrm{H}]}{1 + K_\mathrm{I}[\mathrm{H}]}[\mathrm{I}]_\mathrm{t} \tag{2}$$

$$F = \frac{[\mathrm{I}]_\mathrm{t}}{1 + K_\mathrm{I}[\mathrm{H}]}(f_I + f_\mathrm{HI}K_\mathrm{I}[\mathrm{H}]) \tag{3}$$

Where $[\mathrm{H}]_\mathrm{t}$ = total concentration of the host, [H] = concentration of the host at equilibrium, $[\mathrm{G}]_\mathrm{t}$ = total concentration of the guest, $[\mathrm{I}]_\mathrm{t}$ = total concentration of the indicator, K_G = the association constant of host-guest complex, K_I = the association constant of host-indicator complex, F = measured fluorescence intensity, f_I = the proportionality constant for the indicator, and f_HI = the proportionality constant for the host-indicator complex.

Equation (2) is a cubic equation for [H], which can be rearranged to give equation (4).

$$\mathrm{A}[\mathrm{H}]^3 + \mathrm{B}[\mathrm{H}]^2 + \mathrm{C}[\mathrm{H}] + \mathrm{D} = 0 \tag{4}$$

where

$$\mathrm{A} = K_\mathrm{I}K_\mathrm{G}$$

$$\mathrm{B} = K_\mathrm{I} + K_\mathrm{G} + K_\mathrm{I}K_\mathrm{G}[\mathrm{I}]_\mathrm{t} + K_\mathrm{I}K_\mathrm{G}[\mathrm{G}]_\mathrm{t} - K_\mathrm{I}K_\mathrm{G}[\mathrm{H}]_\mathrm{t}$$

$$\mathrm{C} = 1 + K_\mathrm{I}[\mathrm{I}]_\mathrm{t} + K_\mathrm{G}[\mathrm{G}]_\mathrm{t} - K_\mathrm{I}[\mathrm{H}]_\mathrm{t} - K_\mathrm{G}[\mathrm{H}]_\mathrm{t}$$

$$\mathrm{D} = -[\mathrm{H}]_\mathrm{t}$$

In the assay, we measured the fluorescence intensity of the solution (F_obs) while varying the concentration of the guest ($[\mathrm{G}]_\mathrm{t}$) and keeping the host concentration ($[\mathrm{H}]_\mathrm{t}$) and the indicator concentration ($[\mathrm{I}]_\mathrm{t}$) constant. An experimental isotherm was obtained by plotting F_obs against $[\mathrm{G}]_\mathrm{t}$. The parameters K_I, f_I, and f_HI were determined from the fluorescence titration of the

indicator with the host. The parameter f_I can also be calculated from the fluorescence measurement of a solution containing only the indicator. These known parameters (K_I, f_I, f_{HI}), constants ($[H]_t$, $[I]_t$), variables ($[G]_t$), and the estimated initial value for the unknown parameters (K_G) were used to calculate F_{calc}. The variable [H], needed in the calculation for F_{calc} in equation (3), was determined by solving equation (4) using the Newton–Raphson method. The unknown parameters were then continuously adjusted until the calculated isotherm best fit the experimental one. It is noteworthy that the values of the known parameters were treated as slightly adjustable since they are sensitive to experimental conditions. These iterative processes were achieved through a Python script, as shown below.

```
import numpy as np
import matplotlib.pyplot as plt
from scipy.optimize import curve_fit
import pandas as pd

# Import experimental data
# Load data from CSV file
print('Input file location (full path, including .csv)')
path = input().replace('"', '')  # Replaces all instances of double quotes
with nothing
data = np.genfromtxt(path, delimiter=',', skip_header=1)

# Extract columns into separate arrays
x = data[1:,2]/1000
y = data[1:,3]

# Initialise parameters
F0 = data[0,3] # F when [I] = [I]t (no H and G)
Ht  = data[1,0]/1000
It  = data[0,1]/1000
Ki  = data[0,4]
Kg  = Ki/10
Ei  = F0/It # the proportionality constant for the indicator
a = 1
b = (-1)*(Ht+It+1/Ki)
c = Ht*It
hi = (-b - np.sqrt(b**2 - 4*a*c))/(2*a)
Ehi = Ei - (F0 - y[0])/hi # the proportionality constant for the host-
indicator complex
if Ehi < 0:
    Ehi = y[-1]/It

# Define function
def func(x, Ki, Kg, Ei, Ehi):

    A = Ki*Kg
    B = Ki+Kg+(Ki*Kg*It)+(Ki*Kg*x)-(Ki*Kg*Ht)
    C = 1+Ki*It+Kg*x-(Ki+Kg)*Ht
    D = -Ht
    H = Ht
    step = 1.0
    while np.all(abs(step) > 1e-15):
        step=(A*H*H*H+B*H*H+C*H+D)/(3*A*H*H+2*B*H+C)
        H=H-step # Determine H by Newton-Raphson method
```

```
    return It*(Ei+Ehi*Ki*H)/(1.+Ki*H) # equation (3)

# Perform curve fitting
lowEi = Ei*0.85
highEi = Ei*1.15
lowEhi = Ehi*0.3
highEhi = Ehi*2
bounds = ([19000., 0., lowEi, lowEhi],[23000., 100000., highEi, highEhi])
initial_params = [Ki, Kg, Ei, Ehi]
params, params_covariance = curve_fit(func, x, y, p0 = initial_params,
maxfev = 1000000, bounds = bounds)

# Extract optimized parameters
Ki_fit, Kg_fit, Ei_fit, Ehi_fit = params
sd_value = np.sqrt(np.diag(params_covariance))

# Print the optimized parameters
report = pd.DataFrame({'K(HI;M-1)':[],
                       'K(HG;M-1)':[],
                       'f(I;M-1)':[],
                       'f(HI;M-1)':[]})
report.loc[len(report)] = params
report.loc[len(report)] = sd_value
report.rename(index={0: 'Value', 1: 'SD'}, inplace=True)
print(' ')
pd.options.display.float_format = '{:,.3f}'.format
print(report)

# Generate fitted curve
y_fit = func(x, Ki_fit, Kg_fit, Ei_fit, Ehi_fit)

# Statistic
residuals = y-y_fit
ssr = np.sum(residuals**2)
print('\nss residuals = {:.2e}'.format(ssr))
dof = len(x) - len(params)
chi2_reduced = ssr / dof
print('Reduced chi square = {:5e}'.format(chi2_reduced))
sst = np.sum((y - np.mean(y))**2)
adjusted_r2 = 1 - (ssr / (len(x) - len(params) - 1)) / (sst / (len(x) - 1))
print("Adjusted R-squared = %1.5f" % adjusted_r2)

# Plot the original data and the fitted curve
plt.figure(figsize=(8, 6))
plt.scatter(x, y, label='Data')
plt.plot(x, y_fit, color='red', label='Fitted Curve')
plt.xlabel('Guest concentration / M')
plt.ylabel('Fluorescence')
plt.title('IDA Curve Fitting')

# Remove borders
ax = plt.gca()  # Get current axes
ax.spines['top'].set_visible(False)
ax.spines['right'].set_visible(False)

plt.legend()
plt.show()
```

3. Thermal ellipsoid plot of structure of H•(19)$_{2.7}$

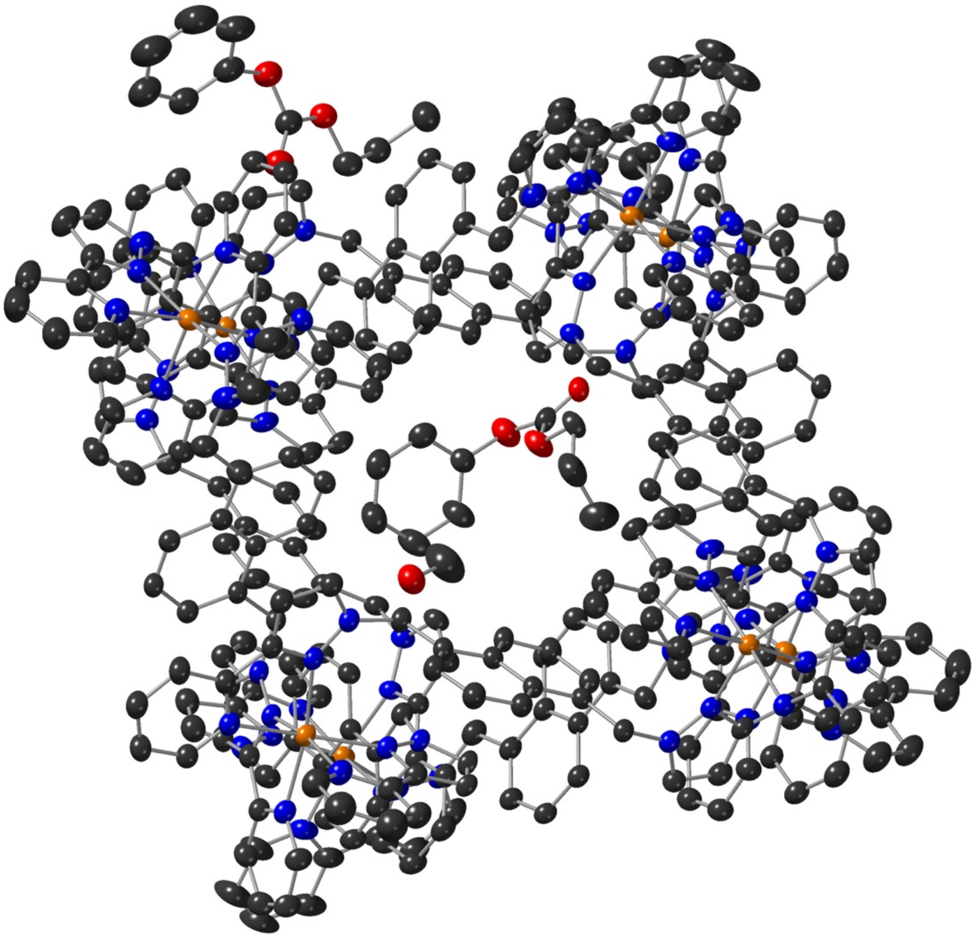

Figure S2. A view based on X-ray crystallography of the host cage **H** (in wireframe view) containing guests **19** (phenyl propyl carbonate) and MeOH, showing thermal ellipsoids at the 40% probability level. An additional guest molecule **19** is located outside the cage cavity.

4. Additional data for reaction progress of the hydrolysis of guests

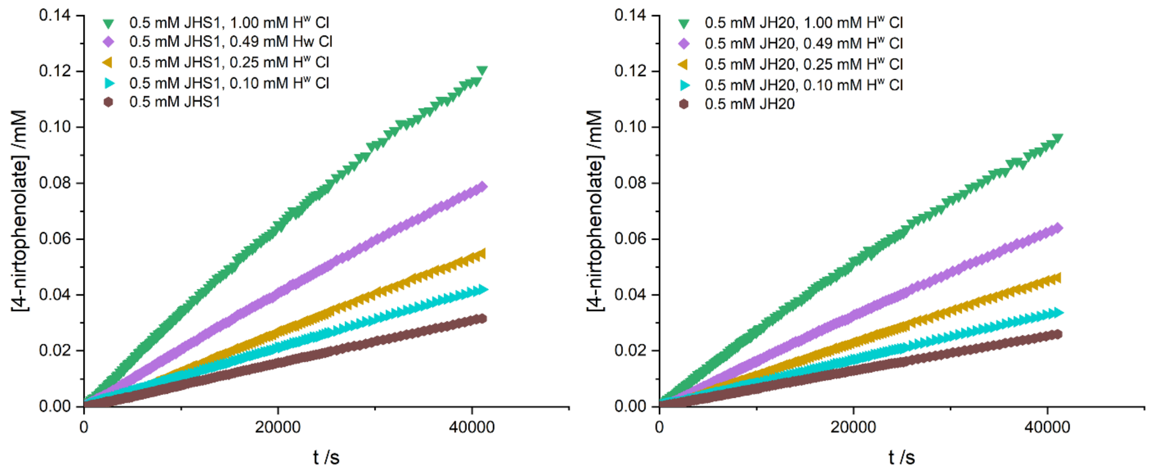

Figure S3. Progress of hydrolysis of guests **20** (left) and **13** (right) at various concentrations of **H^{W}·Cl** under reaction conditions of 2% DMSO/borate buffer (100 mM, pH 9.0) at 303 K. Each data set represents the average of three repetitions.

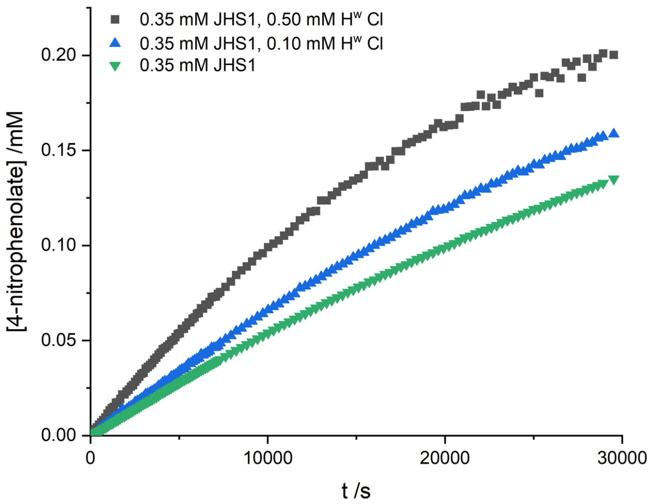

Figure S4. Hydrolysis progress of **20** across different **H^{W}•Cl** concentrations in a 2% DMSO/borate buffer (100 mM, pH 10.0) at 303 K. Data averaged from three repetitions.
